# Chromosome-scale genome assembly of *Codonopsis pilosula* and comparative genomic analyses shed light on its genome evolution

**DOI:** 10.3389/fpls.2024.1469375

**Published:** 2024-11-04

**Authors:** Bao-Zheng Chen, Zi-Jiang Yang, Ling Yang, Yi-Fan Zhu, Xu-Zhen Li, Lei Wang, Ye-Peng Zhou, Guang-Hui Zhang, Da-Wei Li, Yang Dong, Sheng-Chang Duan

**Affiliations:** ^1^ College of Food Science and Technology, Yunnan Agricultural University, Kunming, Yunnan, China; ^2^ Yunnan Provincial Key Laboratory of Biological Big Data, Yunnan Agricultural University, Kunming, Yunnan, China; ^3^ Bioinformatics Group, Wageningen University and Research, Wageningen, Netherlands; ^4^ Institute of Agro-Products of Processing and Design, Hainan Academy of Agricultural Sciences, Haikou, Hainan, China; ^5^ College of Plant Protection, Yunnan Agricultural University, Kunming, Yunnan, China; ^6^ National and Local Joint Engineering Research Center on Germplasm Innovation and Utilization of Chinese Medicinal Materials in Southwest China, Yunnan Agricultural University, Kunming, Yunnan, China

**Keywords:** *Codonopsis pilosula*, assembly, comparative genomics, gene family, genome evolution

## Abstract

**Introduction:**

*Codonopsis pilosula* is a significant plant in traditional Chinese medicine, valued for its edible and medicinal properties. However, the lack of available genomic resources has hindered further research.

**Methods:**

This study presents the first chromosome-scale genome assembly of *C. pilosula* using PacBio CLR reads and Hi-C scaffolding technology. Additionally, Ks analysis and syntenic depth analysis were performed to elucidate its evolutionary history.

**Results:**

The final assembly yielded a high-quality genome of 679.20 Mb, which was anchored to 8 pseudo-chromosomes with an anchoring rate of 96.5% and a scaffold N50 of 80.50 Mb. The genome assembly showed a high completeness of 97.6% based on Benchmarking with Universal Single-Copy Orthologs (BUSCO) analysis. Repetitive elements constituted approximately 76.8% of the genome, with long terminal repeat retrotransposons (LTRs) accounting for about 39.17%. Ks and syntenic depth analyses revealed that the polyploidization history of three platycodonoid clade species involved only the γ-WGT event. Karyotype evolutionary analysis identified an ancestral karyotype with 9 protochromosomes for the three platycodonoid clade species. Moreover, non-WGD genes, particularly those arising from tandem duplications, were found to contribute significantly to gene family expansion.

**Discussion:**

These findings provide essential insights into the genetic diversity and evolutionary biology of *C. pilosula*, aiding its conservation and sustainable use.

## Introduction

The dried roots of *Codonopsis pilosula* (Franch.) Nannf. (Campanulaceae), referred to as “dang shen” in Chinese, are widely used in traditional Chinese medicine. *Codonopsis pilosula* is commonly used as a substitute for the more expensive *Panax ginseng* as a tonic agent, offering comparable therapeutic effects (College, 1986; Meng et al., 2020). Besides its significant medicinal value, *C. pilosula* is also used as a valuable vegetable plant, playing an important role in enhancing dietary nutrition and boosting overall health (He et al., 2016). Previous phytochemical analyses of *C. pilosula* have identified some important secondary metabolites, including triterpenoid saponins, phenylpropanoids, alkaloids, polyacetylenes, and other compounds (Lin et al., 2013; Jiang et al., 2016; Bai et al., 2020; Vo et al., 2024). These findings provide the basis for its pharmaceutical applications, which include anti-tumor (Xu et al., 2012), anti-inflammatory (Meng et al., 2020), immunomodulatory effects (He et al., 2016), anti-fatigue (Cai et al., 2014) and other biological activities (Zou et al., 2020).

Although many studies have contributed to the findings of *C. pilosula*, including the isolation of bioactive compounds (Bai et al., 2020), pharmacological research (Zou et al., 2020), molecular phylogenetic studies (Crowl et al., 2016), and more, the chromosome-level assembly of *C. pilosula* genome has not been reported. The majority of genetic information remains within the nuclear DNA, indicating that the genome-level evolutionary history of *C. pilosula* remains understudied. Furthermore, the genomes of two species within the Campanulaceae family, *C. lanceolata* (Jang et al., 2023) and *Platycodon grandiflorus* (Jia et al., 2022), have been deciphered, offering an excellent genetic resource to investigate their evolutionary relationships and genomic features. Additionally, the progress in genomics, transcriptomics, and related omics fields have greatly enhanced studies in evolutionary and conservation biology. For instance, leveraging one thousand plant transcriptomes, Leebens-Mack et al. (2019) established a strong phylogenomic framework to investigate the evolution of green plants, and their findings suggested that whole-genome duplications have repeatedly taken place during the evolution of flowering plants and ferns. Ma et al. (2022) conducted a study on *Acer yangbiense*, an endangered species with fragmented habitats and a restricted range in Yunnan, China. By resequencing the whole genomes of 105 individuals from the 10 existing populations, they discovered that the species is affected by inbreeding and a significant load of deleterious mutations. These advancements offer greater opportunities for the study of evolution and the conservation of plant resources.

In fact, the natural habitat and population of *C. pilosula* are continuously shrinking, suffering from the worsening climate change and indiscriminate harvesting, which is threatening the sustainable development and genetic diversity of *C. pilosula*. Therefore, generating a high-quality, chromosome-level genome for *C. pilosula* is essential for protecting genetic diversity, understanding the metabolism of its bioactive compound, and providing valuable insights into the evolutionary biology of this significant lineage.

In this study, by combining the PacBio sequencing and Hi-C technology, this effort resulted in a chromosome-level genome assembly of *C. pilosula*. Furthermore, we conducted a comparative genomic analysis on *C. pilosula* with 13 others species. The polyploidization histories of species within the Platycodonoid clade were validated through combined Ks and syntenic depth analyses. The results showed that duplicated genes, especially those resulting from TD and WGD, were the primary factors responsible for gene family expansion. The insights from this study are invaluable for the future conservation and sustainable utilization of this horticulturally and medicinally important plant species.

## Materials and methods

### Plant material and sequencing

Individuals of *C. pilosula* cultivated in the greenhouse of Yunnan Agricultural University were used for sequencing. Fresh leaves were harvested, immediately stored in liquid nitrogen, and subsequently sent to Novogene Bioinformatics Technology Co., Ltd. (Beijing, China) for sequencing. High-molecular-weight DNA was extracted using a modified CTAB method (Allen et al., 2006). The purity and concentration of the extracted DNA were evaluated using 1% agarose gel, and a Qubit fluorometer (Shanghai, China).

For short-read sequencing, a paired-end library was prepared using the NEBNext® Ultra™ Library Prep Kit and sequenced on the Illumina NovaSeq 6000 platform with a read length of 150 bp (Illumina, San Diego, CA, USA). For long-read sequencing, a Continuous Long Read (CLR) SMRTbell library was prepared using the SMRTbell Express Template Prep Kit 2.0 (Pacific Bioscience, CA, USA) according to the manufacturer’s instructions. The long-read sequencing of *C. pilosula* DNA was then conducted on the PacBio Sequel platform (Pacific Bioscience, CA, USA).

A Hi-C library was constructed to generate chromosome-scale assembly. The fresh leaves were collected to construct the Hi-C libraries according to the previous library preparation protocol (Belton et al., 2012). Briefly, samples underwent vacuum infiltration cross-linking for 30 minutes with 3% formaldehyde at 4°C, followed by quenching with 0.375 M glycine for 5 minutes. After lysis of the cross-linked samples, endogenous nucleases were inactivated using 0.3% SDS. Chromatin DNA was then digested with 100 U MboI (New England Biolabs, Ipswich, MA, USA), labeled with biotin-14-dCTP of Invitrogen (Thermo Fisher Scientific, Waltham, MA, USA), and ligated using 50 U T4 DNA ligase (NEB, USA). Cross-links were reversed, and ligated DNA was purified using the QIAamp DNA Mini Kit (Qiagen, Hilden, Germany) following the manufacturer’s instructions. The purified DNA was sheared into 300 to 500 bp fragments and was further blunt-end repaired, A-tailed and adaptor added, followed by purification through biotin-streptavidin–mediated pull-down and PCR amplification. Finally, the Hi-C libraries were quantified and sequenced on the Illumina NovaSeq 6000 platform (Illumina, San Diego, CA, USA) with a mode of paired-end 150 bp.

For protein coding gene annotation, RNA was separately extracted from three tissues (leaf, stem, and root) from the same *C. pilosula* individual using RNAprep pure Plant Kit (TIANGEN, China). RNA libraries were generated using NEBNext^®^ Ultra™ RNA Library Prep Kit for Ilumina^®^ (NEB, USA) following manufacturer’s instruction and sequenced on Illumina NovaSeq 6000 platform. To obtain the transcripts, raw reads were trimmed using Fastp v0.20.1 (Chen et al., 2018) with the following parameters “-q 20 -l 70” and then assembled with Trinity v2.11.0 using the following parameters “–seqType fq –max_memory 100G –CPU 30” (Grabherr et al., 2011).

### Genome assembly and annotation

The genome size was estimated using the Jellyfish v2.0 with the following parameters “-m 17 -s 100G -t 30 -c 7 -C” (Marçais and Kingsford, 2011) and GenomeScope v2.0 with default parameters (Ranallo-Benavidez et al., 2020). To achieve a high-quality genome assembly for *C. pilosula*, we utilized three primary long-read assembly software packages: NextDenovo v2.4.0 (https://github.com/Nextomics/NextDenovo) using the following parameters “task = all; rerun = 3; read_cutoff = 1 k; seed_cutoff = 8 k; seed_cutoff = 8k; genome_size = 683 Mb m; seed_cutfiles = 80; blocksize = 10 g; pa_correction = 80; minimap2_options_raw = -x ava-pb -t 16; sort_options = -m 10g -t 16 -k 50; correction_options = -p 32 random_round = 100 minimap2_options_cns = -x ava-ont -t 20 –k 17 –w 17; nextgraph_options = -a 1”, MaSuRCA v4.1.0 using hybrid mode (Zimin et al., 2013), and Flye v2.9.3-b1797 using a parameter of “–threads 30” (Kolmogorov et al., 2019). The raw assembly was then polished by combining Pacbio CLR reads with Illumina short reads using NextPolish v1.3.1 with default parameters (Hu et al., 2020) for two rounds, followed by the removal of allelic contigs with Purge_Haplotigs v1.1.1 using default settings (Roach et al., 2018). The resulting contigs were scaffolded into chromosome-level scaffolds using Juicer v1.6.2 (Durand et al., 2016) and 3D-DNA pipeline (Dudchenko et al., 2017) with default parameters. To obtain the final genome assembly, the assembly errors (misjoins, misplacements, and orientation errors) in the scaffolds were manually corrected based on Hi-C contact signals using Juicebox v1.13.01 (https://github.com/aidenlab/Juicebox) (Robinson et al., 2018). The quality of the genome was evaluated using BUSCO v5.1.2 (Manni et al., 2021) with dataset embryophyta_odb10 (1,614 BUSCOs). Furthermore, the assembly quality value (QV) was assessed using Merqury v1.4 with a parameter of “k=19” (Rhie et al., 2020).

To identify repeat sequences, we utilized a combination of homology-based predictions and *de novo* predictions. Long Terminal Repeat (LTR) was identified using LTR_FINDER_parallel v1.1 (Ou and Jiang, 2019) with the following parameters “-harvest_out -size 1000000 -time 300 -finder” and LTRharvest v1.0 using the following parameters “-minlenltr 100 -maxlenltr 7000 -mintsd 4 -maxtsd 6 -motif TGCA -motifmis 1 -similar 85 -vic 10 -seed 20 -seqids yes” (Ellinghaus et al., 2008). Subsequently, the LTR candidates were filtered and the LTR Assembly Index (LAI) was calculated using LTR_retriever v2.8 (Ou and Jiang, 2018) with default parameters. Novel repetitive elements were predicted using RepeatModeler v2.0 with a parameter of “-engine rmblast” (Flynn et al., 2020). The predicted repeat libraries from LTR_retriever and RepeatModeler were combined and utilized by RepeatMasker v4.0.9 (http://www.repeatmasker.org) (Tarailo-Graovac and Chen, 2009) for *de novo* prediction with the following parameters “-a -nolow -no_is -norna”. Repetitive elements were annotated with RepeatMasker and RepeatProteinMask v4.0.9 using the parameters of “-engine ncbi -noLowSimple -pvalue 0.0001” with Repbase v24.06 set as database (Bao et al., 2015). Tandem repeats were annotated with Tandem Repeat Finder v4.09 using the parameters of “2 7 7 80 10 50 2000 -d -h” (Benson, 1999). The results from these two annotations were combined to produce the final non-redundant repeat annotation. The chromosomal distributions of Ty3-retrotransposons and Ty1-retrotransposons in *C. pilosula* were calculated using a sliding window of 1 Mbp.

Protein-coding genes were predicted by combining *de novo*, homology-based, and transcript-based methods. Augustus v3.2.2 (Stanke et al., 2008) was used for the *de novo* gene prediction. For the homology-based method, protein sequences of *Arabidopsis thaliana* (L.) Heynh. (GCF_000001735.4) (Willing et al., 2015), *Citrus sinensis* (L.) Osbeck (GCF_022201045.2) (Wu et al., 2023), *Solanum lycopersicum* L. (GCF_000188115.5) (Sato et al., 2012), and *Vitis vinifera* L. (GCF_030704535.1) (Shi et al., 2023) were downloaded from the National Center for Biotechnology Information (NCBI) and aligned to the genome of *C. pilosula* using TBLASTN v2.2.29+ (Camacho et al., 2009) with an E-value threshold of 1e^-5^. GeneWise v2.4.1 (Birney et al., 2004) was then used to predict gene models based on these alignments with default parameters. For the transcripts-based method, the assembled transcripts were mapped to the genome and analyzed using Program to Assemble Spliced Alignments (PASA) v2.4.1 (Haas et al., 2008) to predict genes. Finally, a consensus gene model was combined by EvidenceModeler v1.1.1 (Haas et al., 2008) with the annotated gene structures from *de novo*, homology-based, and transcripts-based methods. Additionally, alternatively spliced sites and untranslated regions (UTRs) were incorporated using PASA v2.4.1 (Haas et al., 2008). For functional annotation of protein-coding genes, we employed eight public databases, including Uniprot, TrEMBL, GenBank NR, KEGG, EggNOG, GO, InterProScan, and Pfam. The annotation process was performed using DIAMOND v0.9.14.115 (Buchfink et al., 2021) with a threshold of e-value ≤ 1e^-5^. The identification of transfer RNAs (tRNAs), was performed using tRNAscan-SE v2.0.7 (Chan et al., 2021). Other non-coding RNAs (ncRNAs), such as microRNAs (miRNAs), ribosomal RNAs (rRNAs), and small nuclear RNAs (snRNAs), were identified using Infernal v.1.1 (Nawrocki and Eddy, 2013) by searching against the Rfam v.14.1 (Kalvari et al., 2021) database.

### Comparative genomics and phylogenetic analyses

The longest protein sequences from *C. pilosula*, along with 13 other species (**Supplementary Table S3**), were clustered into protein sequence in these groups using OrthoFinder v2.5.2 with parameters “-t 30 -a 20 -M msa” (Emms and Kelly, 2019). Single-copy orthologous groups shared by all species were identified and, each protein sequence in these groups was individually aligned using MAFFT v7.475 with parameters “–localpair –maxiterate 1000” (Katoh and Standley, 2013). Subsequently, the corresponding coding sequences (CDS) were aligned to the codon alignments according to the alignments of these protein-coding sequences using PAL2NAL v14 (Suyama et al., 2006). Poorly aligned regions within these codon alignments were filtered out using trimAl v1.4.rev15 with a parameter of “-automated1” (Capella-Gutiérrez et al., 2009). Finally, those codon alignments of the single-copy orthologous groups were concatenated to build a Maximum Likelihood (ML) phylogenetic tree using IQ-TREE v2.2.0.3 with the parameters “-m MFP -bb 1000 -nt 10” and the best-fit model (GTR + F + I + G4) (Nguyen et al., 2015). Divergence times were estimated based on ML tree using MCMCTree v4.10.0 (dos Reis, 2022) from the PAML (Yang, 2007) package with parameters of “burnin=50000; nsample=100000”. Two calibration points were sourced from the TimeTree database (http://www.timetree.org/). The first calibration involved a comparison between *Oryza sativa* and *V. vinifera*, dated between 142 to 163 million years ago (Mya). The second calibration compared *B. vulgaris* with *V. vinifera*, with an estimated divergence time ranging from 111 to 124 Mya. The phylogenetic tree, including the divergence times, was visualized using FigTree v1.4.4 (http://tree.bio.ed.ac.uk/software/figtree/). The gain and loss of orthogroups among 14 species along the phylogenetic tree were estimated using CAFE v5 (Mendes et al., 2021) with a *P* value threshold of 0.05. The GO enrichment analysis of expansion genes was conducted via clusterProfiler v4.2.2 with a *P* value threshold of 0.05 (Wu et al., 2021).

### Synteny and polyploidization exploration

The intraspecific synteny was analyzed using the WGDI toolkit v0.5.1 (Sun et al., 2022). First, intraspecific homologs were extracted using BLASTP v2.2.29+ (Camacho et al., 2009) with an e-value cutoff of 1e^-5^. Collinear gene pairs were identified by WGDI (Sun et al., 2022) with a parameter of ‘-icl’. Ks values of collinear gene pairs were calculated using parameter ‘-ks’ in WGDI with Nei-Gojobori method (Nei and Gojobori, 1986). The medium Ks values of collinear blocks were fitted through Gaussian kernel density estimation by WGDI (Sun et al., 2022) with a parameter of ‘-pf’ and plotted using a parameter of ‘-kf’. The time points of WGD events were calculated according to *T = Ks/2r*, where r represents a substitution rate of 6.5 × 10^−9^ mutations per site per year for eudicots (Tu et al., 2020). The interspecific synteny patterns of *V. vinifera vs*. *C. lanceolata*, *C. lanceolata vs*. *C. pilosula*, and *C. pilosula vs*. *P. grandiflorus* were also investigated by JCVI (Tang et al., 2024). The dot plot between *C. pilosula vs*. *P. grandiflorus* was visualized by WGDI (Sun et al., 2022) with ‘-d’ parameter (Sun et al., 2022). Based on the characterized ancestral karyotype of core eudicots and the inferred polyploidization history (Sun et al., 2022), the common ancestral karyotype between *C. pilosula* and *P. grandiflorus* following the γ-WGT event was illustrated using Adobe Animate software and WGDI (Sun et al., 2022).

### The identification of different modes of gene duplication and the analysis of CYP superfamily

Various gene duplication modes were identified utilizing the “DupGen_finder-unique.pl” module of DupGen_finder (Qiao et al., 2019) with default parameters, and *B. vulgaris* was set as the reference. We identified cytochrome P450 (CYP) and UDP-Glycosyltransferase (UGT) gene family using HMMER v3.1b2 (Potter et al., 2018) with parameter ‘E-value 1e^-5^’. The Pfam HMM models, namely PF00067 and PF00201, were set as queries for the identification of CYP and UGT genes, respectively. The previously characterized *A. thaliana* CYP and UGT genes were downloaded from the database of “The Arabidopsis Cytochrome P450, Cytochrome b5, P450 Reductase, β-Glucosidase, and Glycosyltransferase Site” (http://p450.kvl.dk/index.shtml) and used as outgroups. To construct the phylogenies for CYP genes, the protein sequences were aligned using MAFFT v7.475 (Katoh and Standley, 2013) followed by trimming with trimAl v1.4.rev15 (Capella-Gutiérrez et al., 2009). ML phylogenetic trees were constructed with IQ-TREE v2.2.0.3 (Kalyaanamoorthy et al., 2017) and visualized with the online tool iTOL (https://itol.embl.de/) (Letunic and Bork, 2024).

## Results

### Genome sequencing, assembly, and annotation of *C. pilosula*

**Figure 1:**
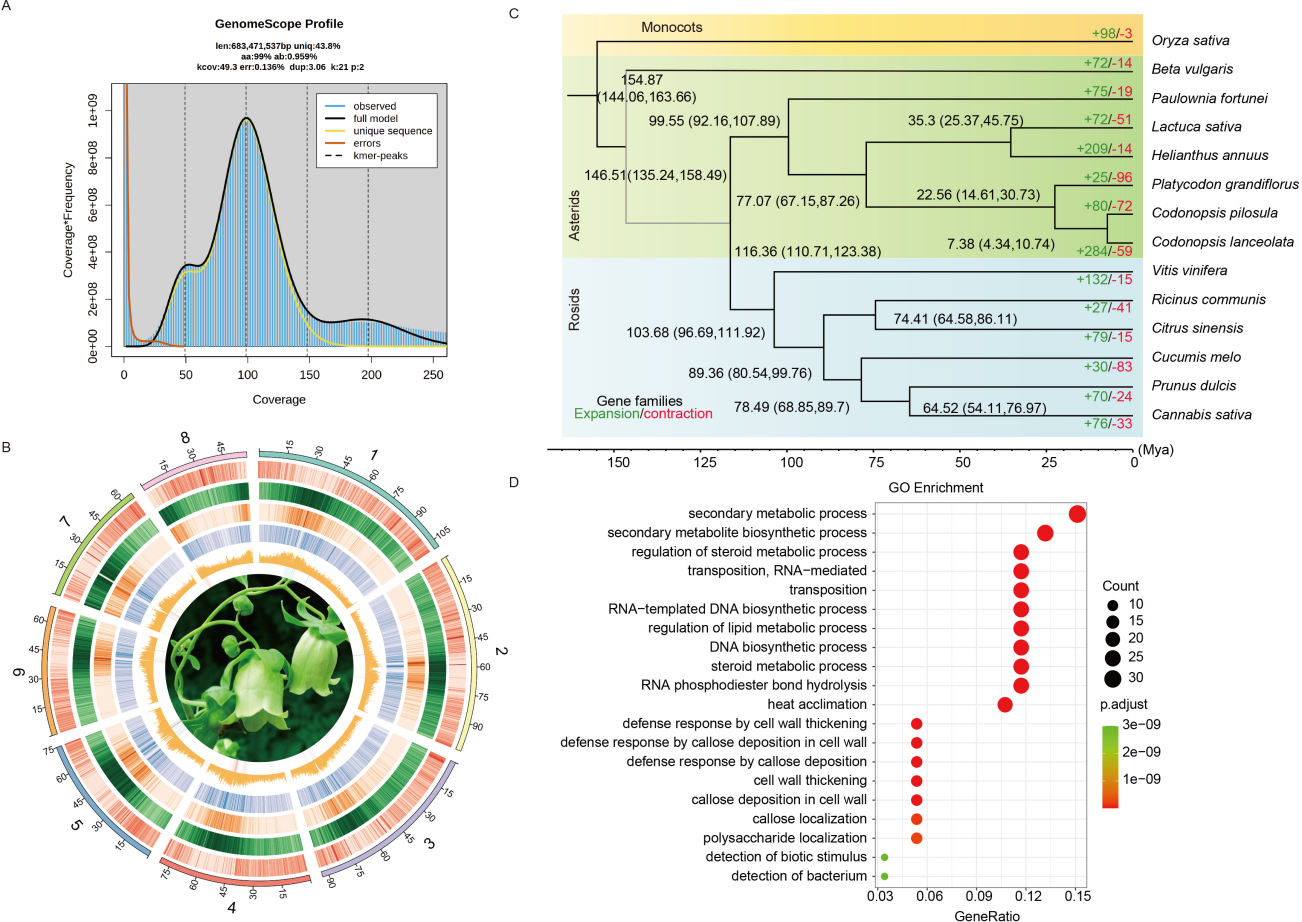
Genomic features and comparative analysis of *C. pilosula* with 13 other species. **(A)** Genome survey of *C. pilosula*. **(B)** The genomic features are arranged in the order of pseudo-chromosomes (scale is in Mb), gene density, repeat density, Ty3-retrotransposons, Ty1-retrotransposons, and GC contents, and flower photos from outside to inside in 1 Mb intervals across the 8 pseudo-chromosomes. **(C)** Phylogenetic tree inferred from single-copy orthologs among selected species. Black numbers in each node denoted the divergence time of each clade (Mya). The green and the red numbers at the terminal branches showed the expansion (green) and contraction (red) of gene families for each species. **(D)** GO enrichment analysis of rapidly expanded genes for *C. pilosula*.

For the assembly of *C. pilosula* genome, we employed long-read PacBio SMRT sequencing, complemented with short-read Illumina NovaSeq 6000 sequencing for error correction. Before assembling the genome, a survey was performed using 80 Gb of Illumina short reads with a K-mer size of 21 (**Supplementary Table S1**). This analysis determined the genome size to be 683.47 Mb and the heterozygosity rate to be 0.96% (**Figure 1A**). In total, 102.77 Gb of PacBio reads (~150 fold genome coverage) were generated for the *de novo* assembly of *C. pilosula* genome (**Supplementary Table S1**). The assembly process using the NextDenovo v2.4.0 generated about 855.06 Mb of sequences with a contig N50 of 2.05 Mb, the MaSuRCA (Hybrid Illumina and PacBio assembly) produced around 712.45 Mb of sequences with a contig N50 of 0.34 Mb, and the Flye generated about 933.67 Mb of sequences with a contig N50 of 0.19 Mb (**Supplementary Table S2**). The BUSCO completeness evaluation revealed that the assembly completeness of all three methods exceeds 97% (**Supplementary Table S2**). Furthermore, based on the results, NextDenovo assembly displayed superior contiguity than other methods, so its primary assembly was selected for further analysis. After two rounds of polishing and the removal of allelic contigs, the resulting draft assembly of *C. pilosula* had a total length of 679.20 Mb and a contig N50 of 2.28 Mb (**Supplementary Table S2**). Next, 96.5% of the draft assemblies were anchored onto eight pseudo-chromosomes in the chromosome-level genome assembly with the 224.89 Gb Hi-C data (~329.26 fold genome coverage) (**Supplementary Table S1**), resulting in a total assembly length of 679.60 Mb and a scaffold N50 of 80.50 Mb (**Figure 1B**; **Supplementary Figure S1**; **Supplementary Table S2**). The size of the final assembled genome was close to the estimations (**Figure 1A**). BUSCO completeness analysis indicated that the final genome assembly contained approximately 97.6% of the embryophyta_odb10 BUSCO gene sets, which is comparable to those of related species (**Supplementary Table S3**). Overall, all Illumina reads were mapped to the *C. pilosula* assembly, achieving a mapping rate of 99.13% and a genome coverage rate of 96.14% (**Supplementary Table S4**). Merqury evaluation revealed that the genome was 91.64% complete with a QV value of 36.50.

For the repetitive elements annotation, the combination of homology-based and *ab initio* predictions revealed that the *C. pilosula* genome contains 70.26% non-redundant repetitive elements. Among these, LTR were the most abundant transposable elements (TEs), constituting 39.17% of the total, while DNA transposons trailed at 8.02% and long interspersed nuclear elements (LINEs) made up 3.77% (**Supplementary Table S5**). Evaluation of the LAI revealed that the genome assembly had an LAI of 14.89, which meets the reference grade suggested by Ou et al. (2018) (**Supplementary Table S5**).

By combining transcriptome-, homology-, and *ab initio*-based techniques, a consensus gene model was generated, resulting in the prediction of 29,808 protein-coding genes, representing 96.50% of the embryophyta_odb10 BUSCO gene sets, which could be comparable with the relative species (**Supplementary Table S6**). Functional annotation indicated that 98.98% of the *C. pilosula* genes could be annotated in at least one of the existing databases, including SwissProt, NR, TrEMBL, KEGG, EggNOG, GO, Pfam, and Interproscan (**Supplementary Table S7**). Furthermore, we identified 912 tRNAs, 345 rRNAs, 137 miRNAs, and 598 snRNAs (**Supplementary Table S8**).

### Phylogenetic and comparative genomics analyses

Based on the clustered results of OrthoFinder v2.5.2, a Maximum Likelihood (ML) phylogenetic tree was constructed using 1,004 single-copy genes present in all 14 species (**Supplementary Table S9**). The analysis revealed that *C. pilosula* was the most closely related to *C. lanceolata* and is also clustered with *P. grandiflorus*. According to the time-calibrated molecular clock, we estimated that Campanulaceae diverged from other Asterids approximately 67.15–87.26 million years ago (Mya), while *C. pilosula* diverged from *P. grandiflorus* approximately 14.61–30.73 Mya (**Figure 1C**).

Among the 29,808 protein-coding genes identified in the *C. pilosula* genome, 27,856 genes were grouped into 15,574 families (**Supplementary Table S10**). Within these families, 773 were unique to *C. pilosula*, while 8,853 families were shared with the other 13 studied species (**Supplementary Tables S9**, **S10**). GO enrichment analysis revealed that these unique paralogous genes were highly enriched in the biological process (BP) terms “DNA replication”, “trichoblast maturation”, and “root hair cell differentiation”; the cellular component (CC) termed “extracellular space” and “nuclear body”; and the molecular function (MF) termed “identical protein binding” and “aminoacylase activity” (**Supplementary Table S11**). Interestingly, some of these genes were closely associated with the development of root hairs.

Gene contraction and expansion analysis showed that, since diverging from *P. grandiflorus*, the *C. pilosula* genome had experienced more gene family expansions (80) than contractions (72) (**Figure 1C**). GO enrichment analysis revealed that the expanded paralogous genes were most enriched in the biological process (BP) terms “secondary metabolic process”, “secondary metabolite biosynthetic process”, and “regulation of steroid metabolic process”; the cellular component (CC) terms “retrotransposon nucleocapsid” and “SCF ubiquitin ligase complex”; and the molecular function (MF) terms “RNA-directed DNA polymerase activity” and “DNA-directed DNA polymerase activity”. Interestingly, these genes are closely associated with steroid and lipid metabolism (**Figure 1D**; **Supplementary Table S12**). In the contracted paralogous gene sets, the GO enrichment analysis revealed that more genes enriched in the biological process (BP) terms “response to ethanol”; the cellular component (CC) terms “lysosome”; and the molecular function (MF) terms “beta-glucosidase activity” (**Supplementary Table S13**).

### The polyploidization and karyotype evolutionary history of platycodonoids clade species

**Figure 2:**
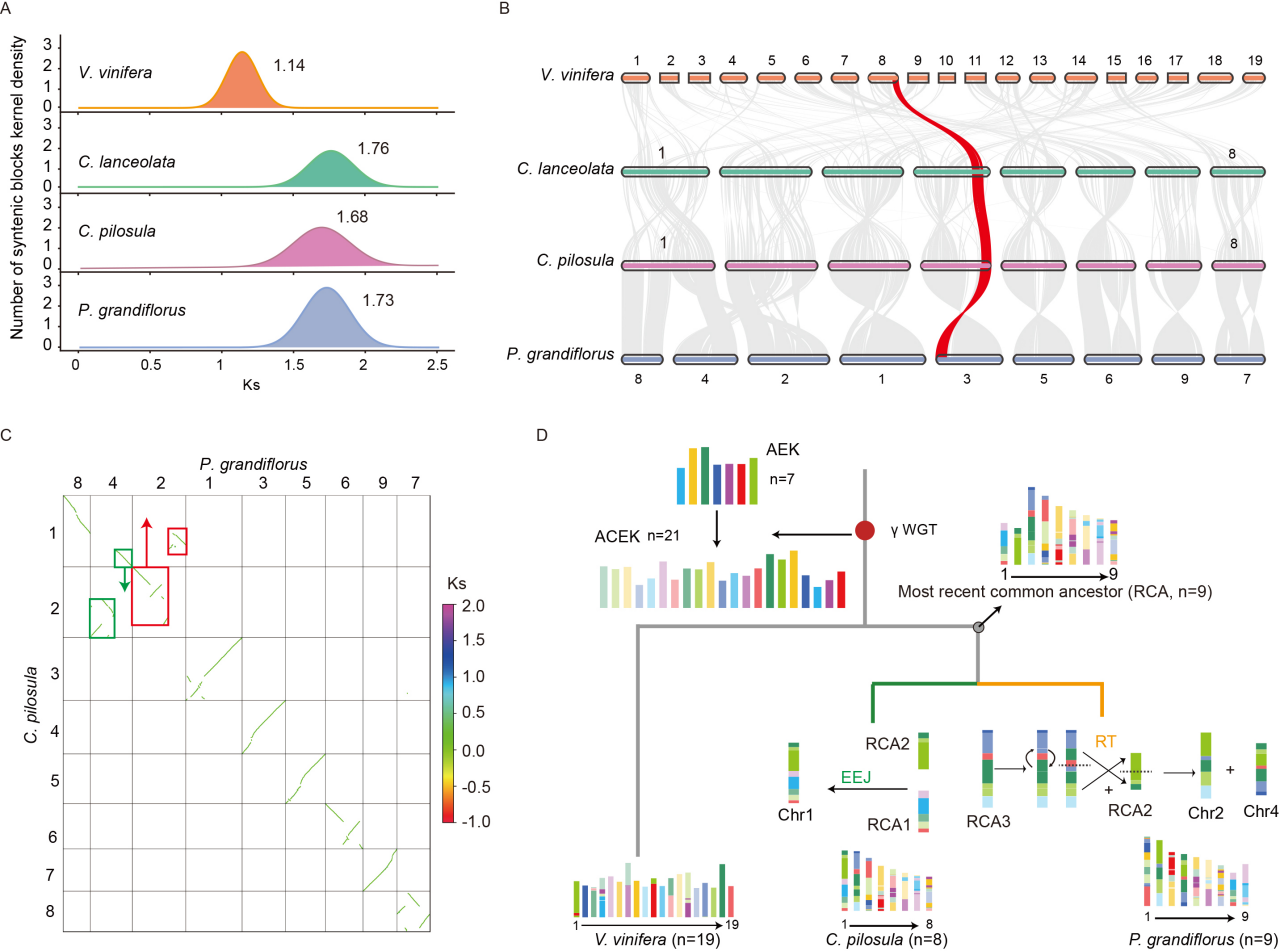
Polyploidization history and karyotype evolution in three platycodonoids species. **(A)** Distribution of the numbers of synonymous substitutions per synonymous site (Ks) of three platycodonoids species and *V. vinifera*. **(B)** The collinearity relationships of three platycodonoids species and *V. vinifera*. **(C)** The green and red boxes represented homologous synteny blocks that underwent reciprocal translocations (RT) in chromosomes 2 and 4 of *P. grandiflorus*. **(D)** Karyotype evolution of *C. pilosula* and *P. grandiflorus*.

To investigate the ancient polyploidization history of platycodonoid clade species in the chromosome-level genome, we analyzed the distribution of substitutions per synonymous site (Ks) in intra-genomic collinear blocks. The Ks analyses revealed that *C. pilosula*, *C. lanceolata*, and *P. grandiflorus* exhibited a single Ks peak, similar to *V. vinifera*, indicating that they all underwent the γ-WGT event shared by all core eudicot species (**Figure 2A**). The distribution of Ks peaks showed a wider range, from 1.14-1.76 (120-135.38 Mya), the lowest Ks value of 1.14 (87.69 Mya) in *V. vinifera* (**Figure 2A**). Among these three platycodonoid clade species, *C. pilosula* exhibited the lowest Ks value of 1.68 (129.23 Mya), representing the slowest evolutionary rate. However, this Ks distribution pattern conflicted with the previous results reported by Crowl et al. (2016), which indicated that *P. grandiflorus* had two Ks peaks at 0.5 and 1.55, representing two WGD events. To further validate this polyploidization history, the analysis of interspecies synteny relationships was conducted. Interspecies synteny comparisons between *V. vinifera* and the three Campanulaceae species suggested that for each *V. vinifera* genomic region there was up to one syntenic region in *C. pilosula*, *C. lanceolata* and *P. grandiflorus*. Syntenic depth comparison of *C. pilosula vs*. *V. vinifera*, *P. grandiflorus vs*. *V. vinifera*, and *C. pilosula vs*. *P. grandiflorus* all exhibited a consistent 1:1 pattern (**Figure 2B**). These analyses confirmed that no additional WGD occurred in these three Campanulaceae species following the γ-WGT.

Synteny analysis revealed that the genomes of *C. pilosula* (x=8) and *C. lanceolata* (x=8) shared the same chromosome numbers and displayed a 1:1 synteny relationship, along with no extra chromosome fusions or fissions (**Figure 2B**). However, the chromosome numbers differed slightly between *C. pilosula* (x=8) and *P. grandiflorus* (x=9). Synteny analysis revealed that six chromosomes in both species might have originated directly from six ancestral chromosomes through several inversions (**Figure 2B**). In contrast, the formation of *C. pilosula* chromosomes 1 and 2 and *P. grandiflorus* chromosomes 2, 4 and 8 was more complicated. Previous studies indicated that chromosomal evolution in land plants was mainly characterized by descending dysploidy, including nested chromosome fusion (NCF) or end-to-end joining (EEJ), as well as non-dysploid changes like inversions, reciprocal translocations (RT), deletions, and duplications (Sun et al., 2022). Based on these findings, we speculated that ancestral chromosomal fusion and non-dysploid changes contributed to the current chromosome structure of *C. pilosula* and *P. grandiflorus*. To infer the likely evolutionary trajectories underlying these chromosomal changes, we visualized the synteny dot plot between them. The *P. grandiflorus* chromosome 8 represented an intact structure of telomere to telomere. Therefore, this intact chromosome was tacked as an ancestral chromosome according to the ‘Telomere-centric genome repatterning model’ proposed in the previous study (Sun et al., 2022) (**Figure 2C**). Then, the synteny relationship between *C. pilosula* chromosomes 1 and 2, and *P. grandiflorus* chromosomes 2 and 4, could be explained by the reciprocally translocated chromosome arms (RTA) (https://github.com/SunPengChuan/wgdi-example/blob/main/Karyotype_Evolution.md) (Sun et al., 2022). However, the history of this RTA is difficult to distinguish according to only these two species. Thus, the interspecies synteny relationships between them and *V. vinifera* were visualized, respectively. Because the *V. vinifera* genome represents the most ancestral eudicot karyotype, it served as the reference (Sun et al., 2022).

Based on the interspecies synteny relationships, the homologous synteny blocks between *V. vinifera* chromosomes 1 and 18, and *P. grandiflorus* chromosomes 2 and 4, were disrupted, whereas this pattern was absent between *V. vinifera* and *C. pilosula* chromosomes 1 and 2 (**Supplementary Figures S2**, **S3**). Furthermore, *V. vinifera* chromosomes 18 was previously proved to represent an intact ancestral chromosome of the eudicot karyotype (Sun et al., 2022). To further verify if the results were due to assembly errors, a collinear analysis was performed using data from Jia et al. (2022) and Lee et al. (2023). This analysis showed that homologous chromosomes, especially chromosomes 2 and 4, had stronger collinear relationships, indicating no significant assembly errors (**Supplementary Figure S4**). Therefore, according to the ‘Telomere-centric genome repatterning model’, we believed that the *P. grandiflorus* chromosomes 2 and 4 experienced a RT, rather than the *C. pilosula* chromosomes 1 and 2. Based on those findings, we inferred that platycodonoids clade species had a common ancestral karyotype n=9 (**Figure 2D**).

### Repetitive elements driving the evolution of the genome

**Figure 3:**
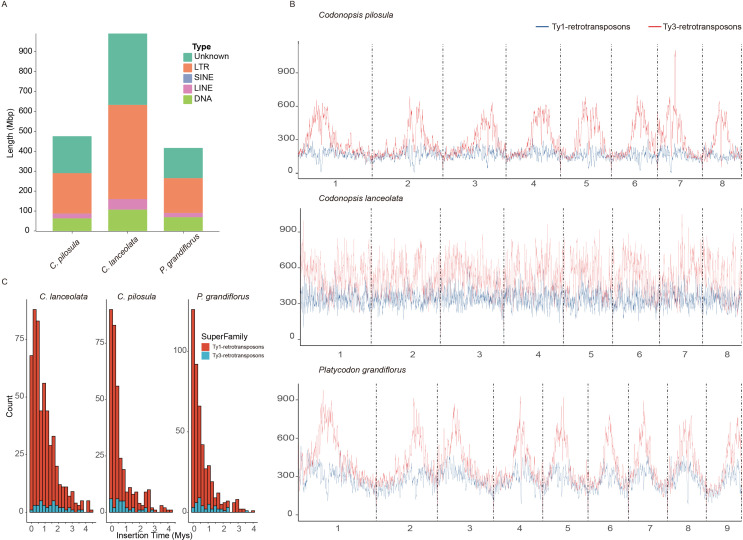
Evolutionary analysis of TEs in three platycodonoids species. **(A)** Sequence size statistics for various types of transposable elements (TEs). **(B)** LTR density distribution. The Ty1-retrotransposons indicated by a blue line and the Ty3-retrotransposons indicated by a red line. **(C)** Insertion time distribution of intact Long Terminus Repeat (LTR).

Generally, the genome size variation could be largely attributed to the difference of the transposable elements (TEs) or the polyploidization events (Kapusta et al., 2017; Wang et al., 2021). *C. pilosula*, *C. lanceolata*, and *P. grandiflorus* shared a common polyploidization history according to the previous results in this study. However, the genome size of *C. pilosula* (679.60 Mb) is 9.05% larger than that of *P. grandiflorus* (622.86 Mb) and appreciably smaller by 46.62% compared to *C. lanceolata* (1,273.24 Mb) (**Supplementary Table S3**). Therefore, to reveal the causes for the variation in genome size among these three species, comparative analyses of their TEs evolution and contents were conducted. This analysis indicated that *C. lanceolata* had the highest TE content of 950.72 Mb (74.67%) among these three species. (**Figure 3A**; **Supplementary Table S14**). The *C. pilosula* and *P. grandiflorus* showed similar TE contents. LTR elements were the most abundant types among the classified TEs, constituting over 28% of the three species (**Figure 3A**; **Supplementary Table S14**).

To gain a clear understanding of the landscape of TEs in these three species, the chromosomal distribution of LTR elements, specifically Ty3-retrotransposons and Ty1-retrotransposons, was visualized. The abundance of Ty3-retrotransposons was higher than that of Ty1-retrotransposons in these three species. A comparable content of TEs was observed in the genomes of *C. pilosula* and *P. grandiflorus*, consistent with their TE contents (**Figure 3B**). The Ty3-retrotransposons and Ty1-retrotransposons were much more abundant in the pericentromeric regions compared to the chromosomal terminal regions in both species. However, this high density TE distribution in pericentromeric regions was not evident in the *C. lanceolata* genome (**Figure 3B**). This pattern was uncommon in the genomes of most reported species. Meanwhile, both Ty3-retrotransposons and Ty1-retrotransposons in *C. lanceolata* showed higher abundance compared to *C. pilosula* and *P. grandiflorus* (**Figure 3B**). Therefore, this higher TE contents were responsible for the expansion of genome size indeed.

We further estimated the insertion times of LTR in these three species. The insertions of the majority of the Ty1-retrotransposons started at ~0-1.5 Mya for the three species. The proliferation of Ty1-retrotransposons in *C. pilosula* and *P. grandiflorus* exhibited a similar pattern, peaking around 0.1 Mya, slightly earlier than *C. lanceolata* at ~0.25 Mya. It is noted that *C. pilosula* and *P. grandiflorus* had an obvious Ty1-retrotransposons proliferation at ~2.5 and ~2.9 Mya, respectively. Moreover, the majority of Ty3-retrotransposons insertions at 0.5 Mya in *C. pilosula* and *P. grandiflorus*, which lagged behind the peaks of Ty1-retrotransposons. In *C. lanceolata*, the major insertions of Ty3-retrotransposons LTR occurred around at 0.5-2Mya. This higher density of insertions may explain its greater Ty3-retrotransposons content compared to the other two species in platycodonoid clades. *C. pilosula* also showed a minor increase in Ty3-retrotransposons proliferation around ~2.25 Mya, while this phenomenon was absent in the other two species (**Figure 3C**).

### Duplicated genes driving the expansion of gene families

**Figure 4:**
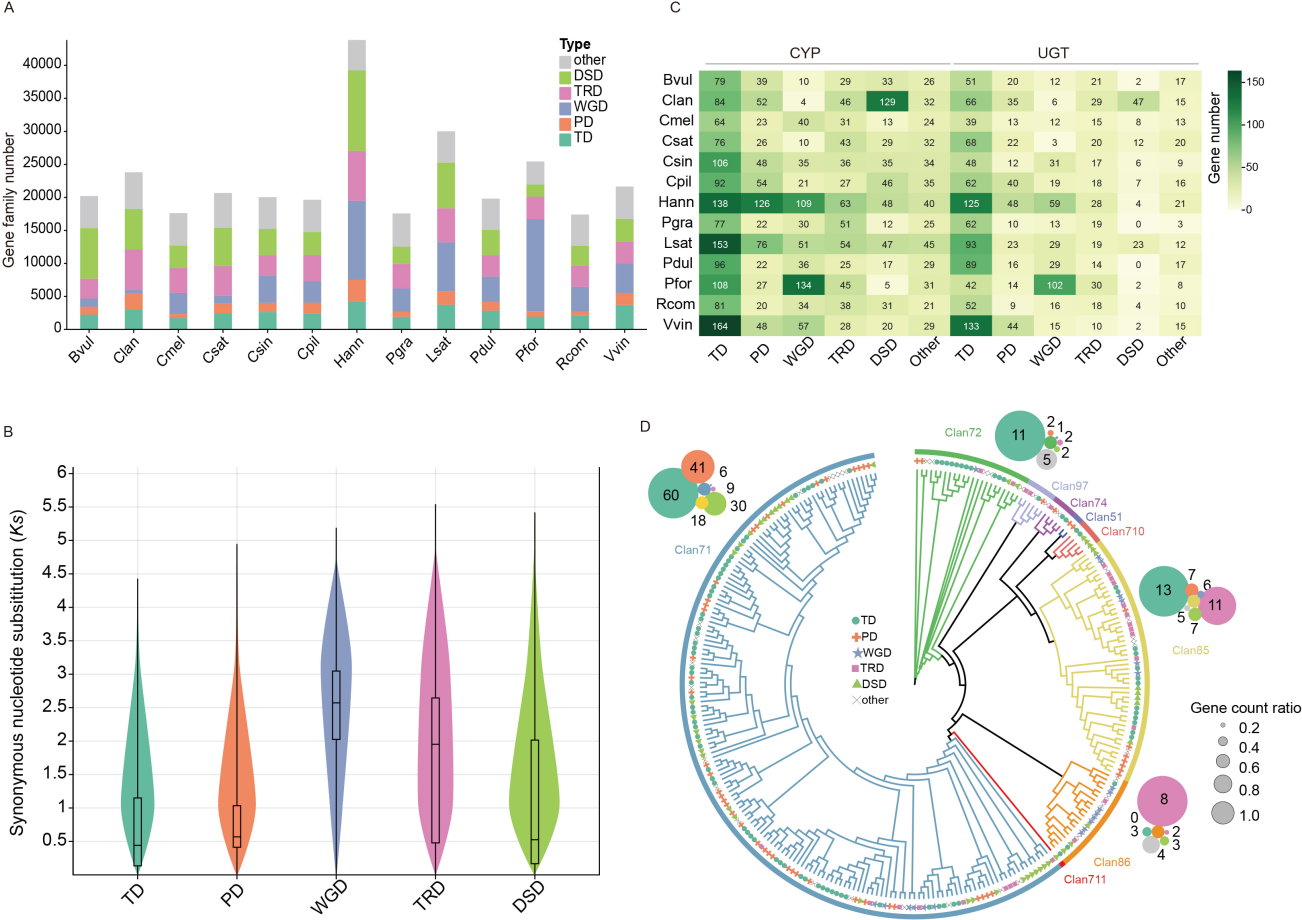
The numbers and distributions of various duplicated genes across 13 species. **(A)** The distribution of transposed duplicates (TRD), dispersed duplicates (DSD), tandem duplicates (TD), proximal duplicates (PD), whole-genome duplicate (WGD) and other genes. Bvul (*B. vulgaris*), Clan (*C. lanceolata*), Cmel (*C. melo*), Csat (*C. sativa*), Csin (*Citrus sinensis*), Cpil (*C. pilosula*), Hann (*Helianthus annuus*), Pgra (*Platycodon grandiflorus*), Lsat (*Lactuca sativa*), Pdul (*Prunus dulcis*), Pfor (*Paulownia fortune*), Rcom (*Ricinus communis*), Vvin (*Vitis vinifera*). **(B)** The distribution of synonymous substitutions per synonymous site (Ks) in *C. pilosula* for various modes of gene duplications. **(C)** The proportions of cytochrome P450 (CYP) and UDP-Glycosyltransferase (UGT) genes from different gene duplications. **(D)** The phylogenetic tree and the proportions of 9 Clan CYP genes from *C. pilosula*. The outer circles represent the counts of gene duplication modes for the four largest Clan CYP genes: Clan 71, Clan 85, Clan 72, and Clan 86.

WGD events and duplicated genes are the primary driving force for the gene family expansion (Magadum et al., 2013). Generally, duplicated genes could be classified into five types including whole-genome duplication (WGD), tandem duplication (TD), proximal duplication (PD), dispersed duplication (DSD), and transposed duplication (TRD) (Qiao et al., 2019). Genes with the same Pfam domain are typically grouped into the same gene family. Therefore, to investigate the influence of duplicated genes onto the expansion of gene families, the genes with the Pfam annotation were classified into different duplicated gene types across the analyzed 13 species. This analysis showed that over 72% of the Pfam annotated genes could be classified into five duplicated gene types among these 13 species (**Figure 4A**; **Supplementary Table S15**). This higher proportion suggested that duplicated genes indeed played important roles in driving the expansion of gene families. Interestingly, *Helianthus annuus*, *Lactuca sativa*, and *Paulownia fortunei* showed a higher proportion of WGD genes compared to other species, due to their history of additional WGD events, with the exception of the γ-WGT event (**Figure 4A**). The Ks analyses of duplicated genes in *C. pilosula* showed that WGD and TRD genes exhibited a higher average Ks value distribution, resembling the Ks peak value of the γ-WGT event (**Figures 2A**, **4B**). The Ks average value of TD, PD, and DSD genes were all less than 1, indicating that most of them could have been generated following the γ-WGT event (**Figure 4B**).

To illustrate the impact of duplicated genes in more details, two major gene families, CYP and UGT, were selected for the study. The distribution of duplicated genes in these two gene families notably differed from that of the total genes across the 14 species. The TD genes showed the highest level of abundance, accounting for almost 1/3 of the CYP or UGT gene families across the 13 species, respectively (**Figure 4C**). It is worth noting that the PD and DSD genes also had a significant proportion in the *Helianthus annuus* and *C. lanceolata* CYP genes. Interestingly, although 10 out of the 13 species shared a common polyploidization history with the γ-WGT event, they exhibited a relatively low proportion of WGD genes in their CYP and UGT gene families. In contrast, the other three species, *H. annuus*, *L. sativa*, and *P. fortune*, which have undergone additional recent WGD events, showed a higher proportion of WGD genes in these gene families (**Figure 4C**). This phenomenon may be attributed to the loss or neofunctionalization of the majority of duplicated genes generated from γ-WGT event during the long evolutionary history. To investigate the duplicated genes distribution of CYP gene family in different clans, the phylogenetic tree of *C. pilosula* CYP genes was constructed. Totally, 275 CYP gene sequences were further clustered into nine clans. Clan711 is single-family CYP clan. Clan71 is the largest CYP clan, followed by Clan85, Clan72, Clan86, Clan97, Clan710, Clan74, and Clan51. Clan71 comprised over half of all genes and covered all types of duplicated genes (**Figure 4D**). Among them, four larger clans, namely Clan71, Clan85, Clan72, and Clan86, were selected to investigate the distribution of duplicated genes. The analysis of the duplicated genes showed that over 1/3 of Clan71 CYP genes originated from TD genes, followed by PD, DSD, other, TRD, and WGD. Within Clan85 and Clan72, TD genes retained their position as the most prevalent. Conversely, Clan86 showcased the highest proportion of WGD genes (**Figure 4D**).

## Discussion

The completion of the chromosome-level genome assembly for *C. pilosula* marks a significant advancement in deciphering the genomic intricacies of this species. The integration of multiple sequencing technologies and various assembly strategies has resulted in a comprehensive genomic resource. This lays the groundwork for in-depth studies of the *C. pilosula* genetic landscape. Rigorous validation steps, such as mapping Illumina short reads, LAI index, and BUSCO evaluations, have collectively affirmed the reliability, accuracy, and completeness of the *C. pilosula* genome assembly (**Supplementary Tables S2**, **S5**). These metrics underscore the technical robustness of the assembly process and provide confidence for subsequent analyses and interpretations. The complete *C. pilosula* genome assembly enhances our understanding of both the coding and non-coding components of the genome, which is important for future studies on gene regulation and functional genomics.

By integrating the annotated *C. pilosula* genome with previously published genomes representing Campanulaceae members, we identified one round of WGD event (γ-WGT) in *C. pilosula*, *C. lanceolata*, and *P. grandiflorus* (**Figure 2A**). The observed collinearity between the genome, along with comparisons to other species, strongly supported this γ-WGT event (**Figures 2B, C**). However, this finding contradicts the previous two rounds of WGD events proposed by Crowl et al. (2016), which were only inferred from transcriptome data. Generally, WGD inference based on Ks values was limited due to the difficulty in distinguishing gene pairs originating from WGD and those arising from small-scale duplications (SSDs) without structure information (Zwaenepoel et al., 2018). For example, some misinterpretations of the correspondence between Ks values and WGDs based on transcriptome alone had been demonstrated in some species, such as *Callicarpa americana* (Hamilton et al., 2020), watermelon (Guo et al., 2013), black pepper (Hu et al., 2019), Olive (Ren et al., 2018), and *Prunus mongolica* (Zhu et al., 2023). In summary, integrating genomic collinearity analysis with Ks information provided a more accurate and effective method for inferring polyploidization events, as supported by our findings in this study and previous research (Kong et al., 2023; Sun et al., 2024).

Following the WGD events, numerous species underwent the post-polyploid diploidization (PPD) processes, as evidenced in various specific lineages (Mandáková and Lysak, 2018). PPD processes mainly involve changes in genome size, chromosomal rearrangements, subgenome-specific fractionation, including biased gene retention/loss and gene sub-/neofunctionalization, and others (Cheng et al., 2018; Mandáková and Lysak, 2018). Among them, chromosomal rearrangements, including EEJ, NNC and RT (Sun et al., 2022), represented the most dramatic type of karyotype evolution, injecting significant potential for the species diversity and speciation. According to the ‘Telomere-centric genome repatterning model’ proposed in the previous study (Sun et al., 2022), the platycodonoids clade species had a common ancestral karyotype n=9 (**Figure 2D**). Subsequently, *C. pilosula* and *C. lanceolata* underwent a single EEJ fusion to form the present karyotype n=8; *P. grandiflorus* experienced a single RT without any additional chromosome fusion events to achieve the current karyotype n=9 (**Figure 2D**). This *Codonopsis*-specific EEJ fusion could play a crucial role in driving the speciation of *Codonopsis* and *Platycodon*.

In the plant kingdom, species vary in genome size from a few hundred Mbs to tens of gigabytes (Pellicer et al., 2018). This variation is typically attributed to factors such as polyploidization, gene duplications, repeat expansion, and other events (Wendel et al., 2016; Wang et al., 2021; Zuntini et al., 2024). In our study, the findings indicated that three platycodonoid clade species has only undergone the γ-WGT event. The shared polyploidization history of these species provides ideal materials for a deeper exploration of the mechanisms underlying their unique genomic expansion. Our analyses of repetitive elements indicated these repetitive elements play an important role in driving genome expansion and causing variations in genome size. The significance of repetitive elements in driving genome size has also been confirmed in other species, such as hawthorn (Zhang et al., 2022), *Welwitschia* (Wan et al., 2021), and *Cycas* (Liu et al., 2022). LTR elements account for almost half of the repetitive elements. The analyses of insertion times indicated that the proliferation of LTR elements varied among these three species. Interestingly, many LTR elements burst took place after their speciation, suggesting that this differing proliferation could be derived from their adaptive evolution. However, this adaptive assumption is awaiting more conclusive evidence.

Polyploidization and gene duplication have been important driving forces of genome evolution, playing a crucial role in adaptation to new environments (Liu et al., 2023; Ye et al., 2024). Our analyses of different duplication modes in gene families revealed that WGD, TD, PD, DSD, and TRD have profound implications for the expansion of gene families, with TD exhibiting the most obvious impact. WGD also apparently influenced the gene families of *H. annuus*, *L. sativa*, and *P. fortune* as they all underwent additional WGD events following the γ-WGT event. In contrast, this phenomenon is less apparent in the other 11 species, which only experienced the γ-WGT event, possibly due to the drastic loss of most WGD genes over their long evolutionary history (Gout et al., 2023). Furthermore, two specific gene families, CYP and UGT, were included in this analysis. The results showed that many genes tend to be clustered within TD, further indicating its important contributions to gene family evolution. In summary, polyploidization and other duplicated genes play important roles in driving the evolution of gene families, commonly expanding them. However, the dynamic influence on gene families, as well as the corresponding gene fate, remain understudied topics.

In conclusion, this study presents the first high-quality haploid genome assembly for *C. pilosula*. This marks the first step towards understanding the molecular basis of various desirable traits in economically important species. Although more research is needed on chromosomal structural diversity and haplotype-resolved genomes, our findings provide a basis for future research in comparative genomics, molecular biology, genetics, and evolutionary aspects of species of the platycodonoid clade.

## Data Availability

The raw genome sequencing data of *C. pilosula* are available at the National Center for Biotechnology Information (NCBI) under BioProject number PRJNA1068481. The raw sequence and genome sequence (https://ngdc.cncb.ac.cn/gwh/Assembly/85966/show, GWHFAJJ00000000.1) have also been uploaded to NGDC database under the accession of PRJCA029124 (CRA018369). Besides, the annotation results, including protein coding genes and repeat annotation, have been uploaded to the Figshare database (https://doi.org/10.6084/m9.figshare.26799025). All data are available from the corresponding author upon request.
