# Supplementary material for: Chromosome-scale genome assembly of Codonopsis pilosula and comparative genomic analyses shed light on its genome evolution
Source: Front Plant Sci. 2024 Nov 4;15:1469375. doi: 10.3389/fpls.2024.1469375 (PMC11570261; doi:10.3389/fpls.2024.1469375)
Supplement: Supplementary file 1 [file DataSheet1.pdf]

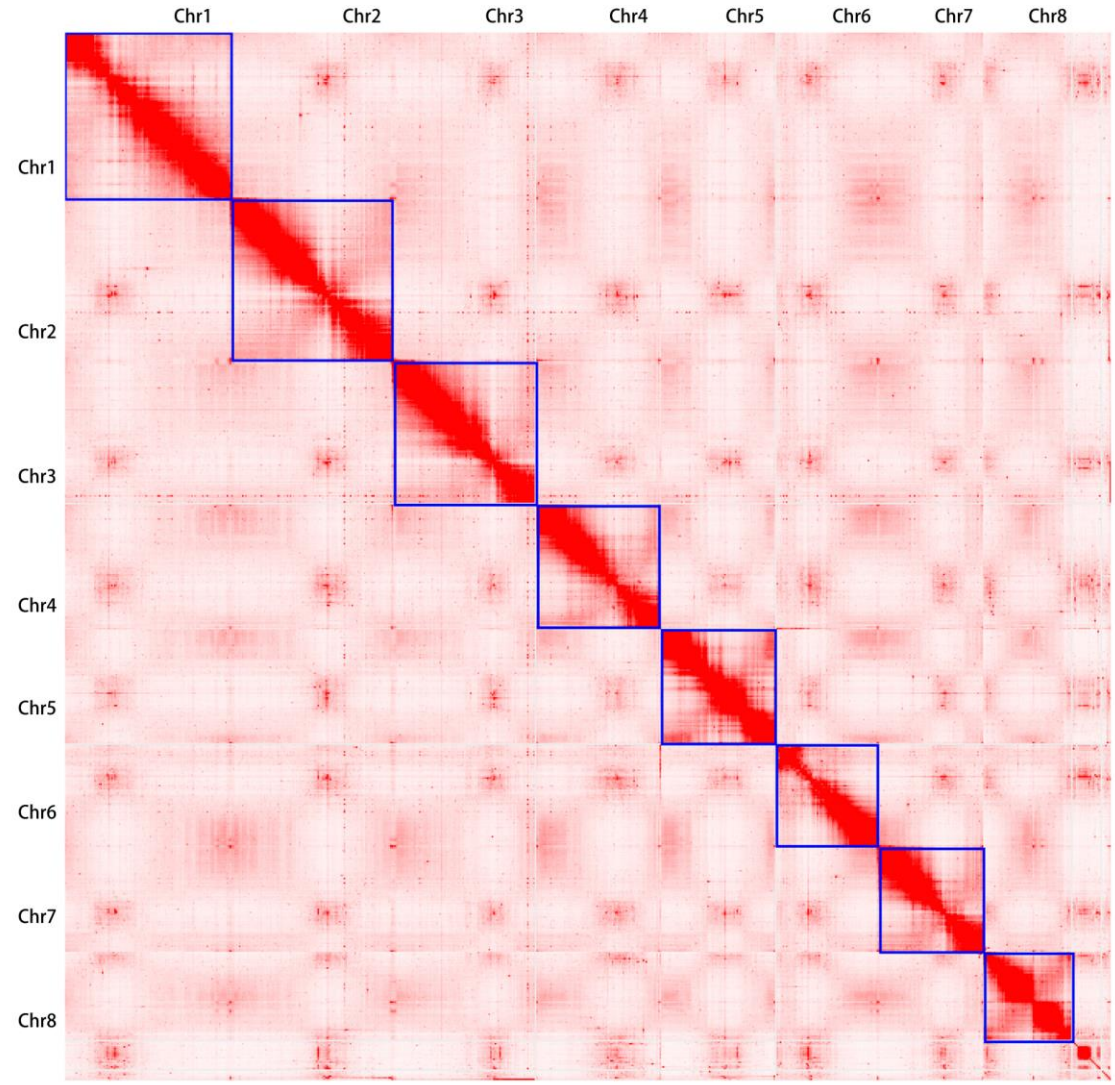

Fig. S1. Hi-C interaction heatmap and overview of the *C. pilosula* genome with a resolution of 500 kb. The blue color of each square indicates the borders between scaffolds.

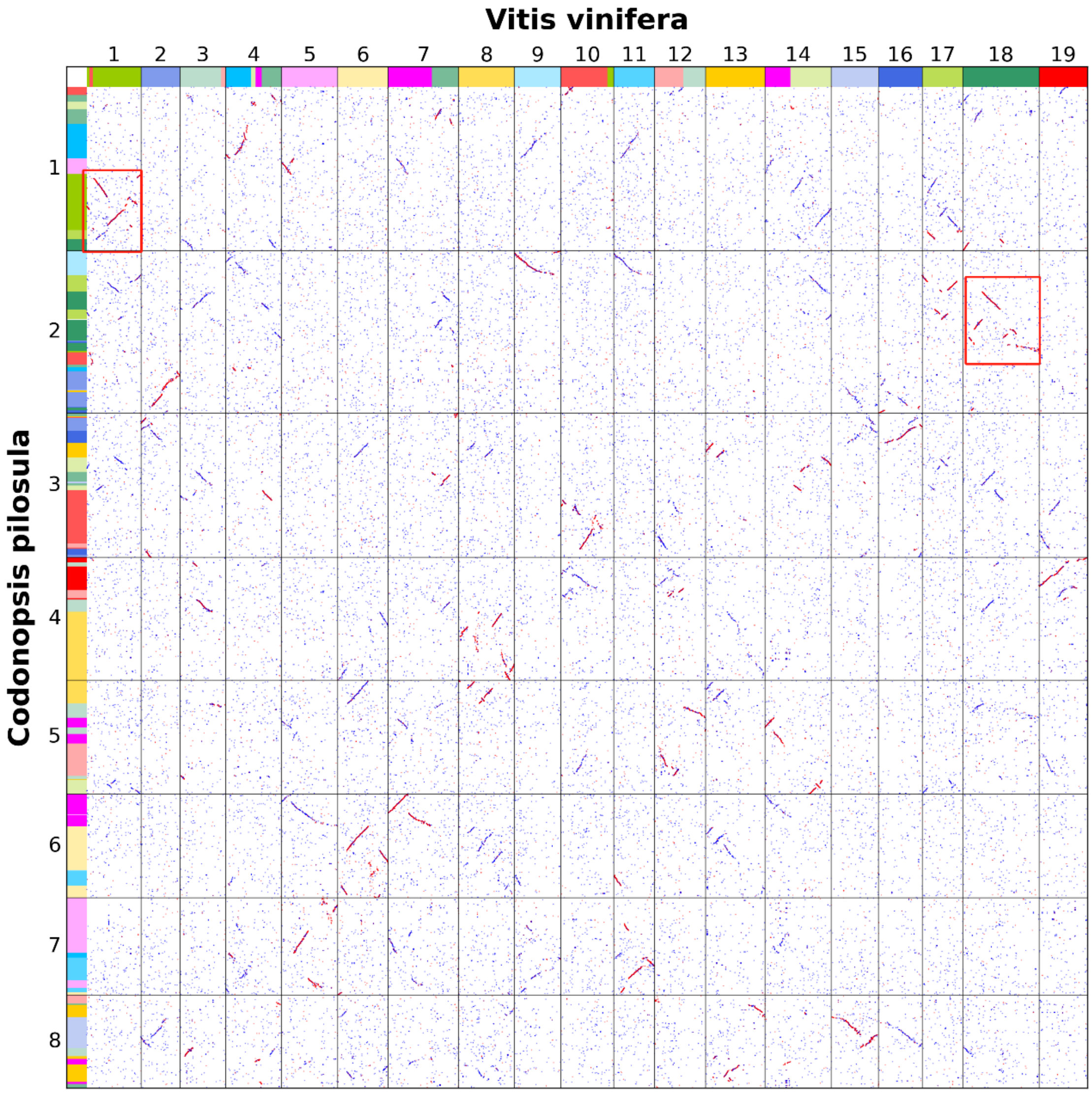

Fig. S2. Syntenic dot plot between *C. pilosula* and *V. vinifera*. If the anchor gene pairs were the best BLAST hits among the genomes, they were plotted as red dots; otherwise, they were shown in blue dots. The red box indicated that the homologous synteny blocks between *V. vinifera* chromosomes 1 and 18, and *C. pilosula* chromosomes 1 and 2, were not disrupted.

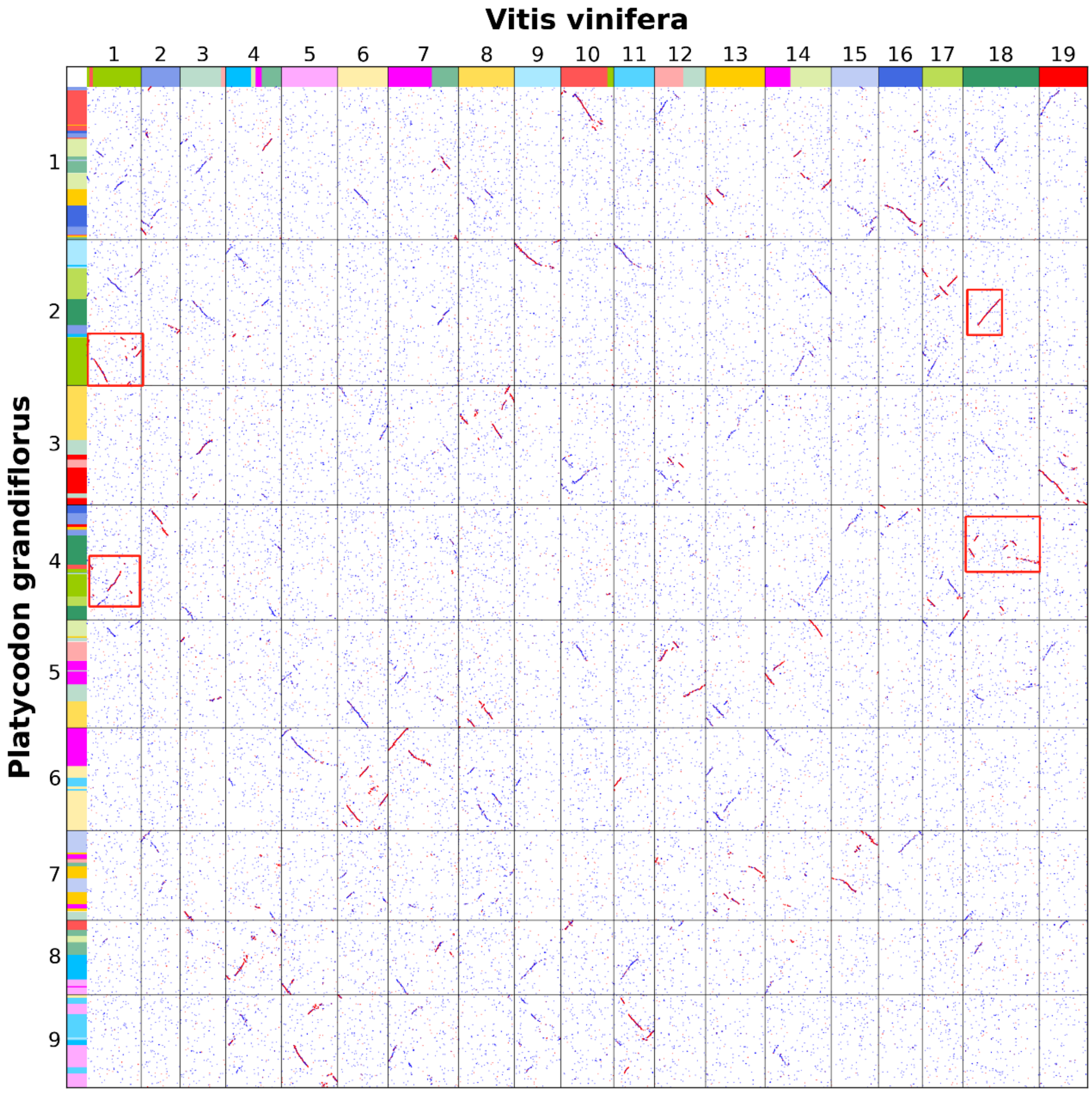

Fig. S3. Syntenic dot plot between *P. grandiflorus* and *V. vinifera*. If the anchor gene pairs were the best BLAST hits among the genomes, they were plotted as red dots; otherwise, they were shown in blue dots. The red box indicated that the homologous synteny blocks between *V. vinifera* chromosomes 1 and18 and *P. grandiflorus* chromosomes 2 and 4 were disrupted.

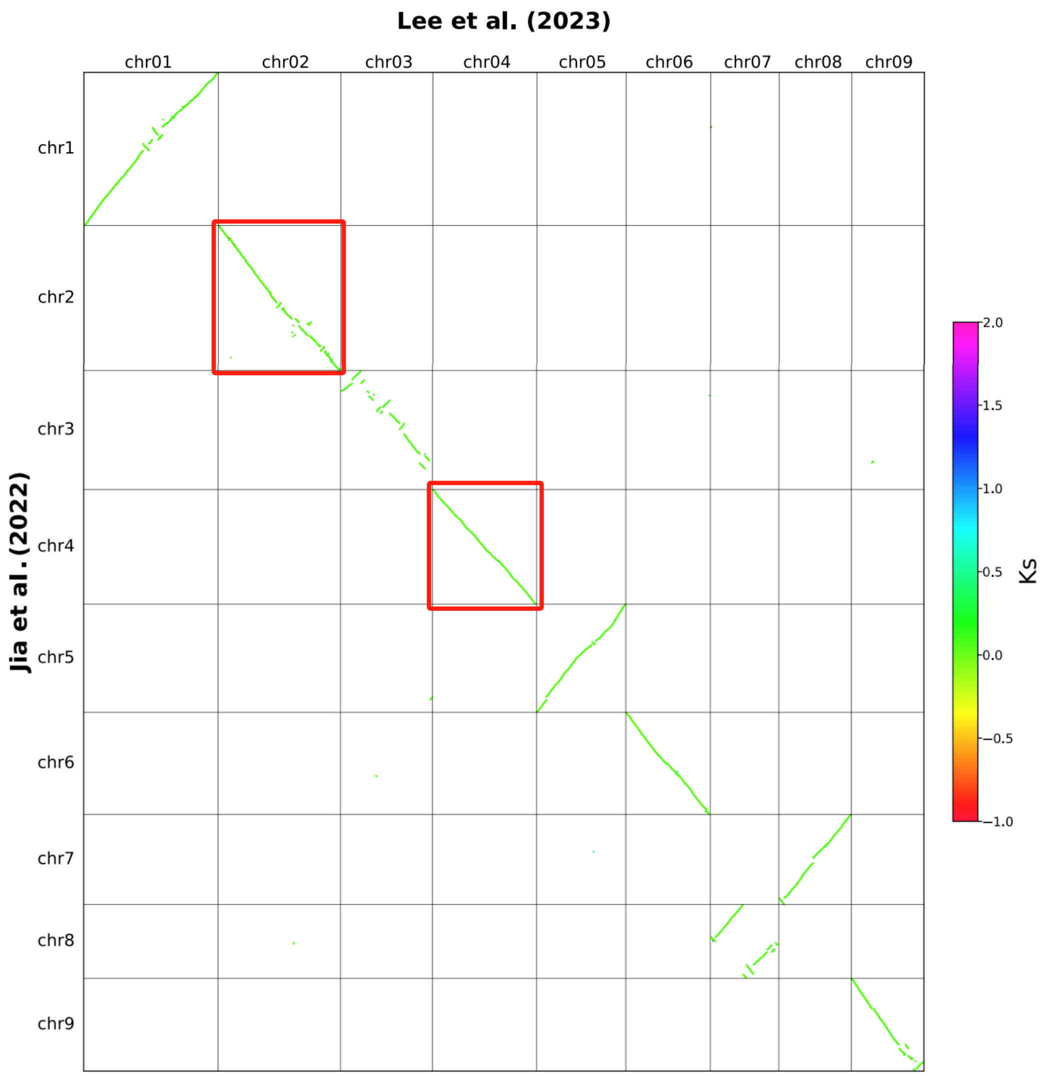

Fig. S4. Syntenic dot plot between the *P. grandiflorus* genome assemblies by Jia et al. (2022) and by Lee et al. (2023). The red box indicated the collinear relationships of chromosomes 2 and 4.

Jia, Y., Chen, S., Chen, W., Zhang, P., Su, Z., Zhang, L., et al. (2022). A Chromosome-Level Reference Genome of Chinese Balloon Flower (Platycodon grandiflorus). *Front Genet* 13**,** 869784. doi: 10.3389/fgene.2022.869784.

Lee, D.-J., Choi, J.-W., Kang, J.-N., Lee, S.-M., Park, G.-H., and Kim, C.-K. 2023. Chromosome-Scale Genome Assembly and Triterpenoid Saponin Biosynthesis in Korean Bellflower (Platycodon grandiflorum). *International Journal of Molecular Sciences* [Online], 24(7).
